# Malate-Dependent Carbon Utilization Enhances Central Metabolism and Contributes to Biological Fitness of *Laribacter hongkongensis* via CRP Regulation

**DOI:** 10.3389/fmicb.2019.01991

**Published:** 2019-08-28

**Authors:** Lifeng Xiong, Elaine Chan, Jade L. L. Teng, Siguo Liu, Susanna K. P. Lau, Patrick C. Y. Woo

**Affiliations:** ^1^Division of Bacterial Diseases, State Key Laboratory of Veterinary Biotechnology, Harbin Veterinary Research Institute, Chinese Academy of Agricultural Sciences, Harbin, China; ^2^Department of Microbiology, Li Ka Shing Faculty of Medicine, The University of Hong Kong, Hong Kong, Hong Kong; ^3^Research Centre of Infection and Immunology, The University of Hong Kong, Hong Kong, Hong Kong; ^4^State Key Laboratory of Emerging Infectious Diseases, The University of Hong Kong, Hong Kong, Hong Kong; ^5^Carol Yu Centre for Infection, The University of Hong Kong, Hong Kong, Hong Kong; ^6^Collaborative Innovation Center for Diagnosis and Treatment of Infectious Diseases, The University of Hong Kong, Hong Kong, Hong Kong

**Keywords:** *Laribacter hongkongensis*, malate, metabolism, biological fitness, CRP, regulation, adaptation

## Abstract

Metabolic adaptation in various environmental niches is crucial for bacterial extracellular survival and intracellular replication during infection. However, the metabolism of carbon/nitrogen sources and related regulatory mechanisms in *Laribacter hongkongensis*, an asaccharolytic bacterium associated with invasive infections and gastroenteritis, are still unknown. In the present study, we demonstrated that malate can be exploited as a preferred carbon source of *L. hongkongensis*. Using RNA-sequencing, we compared the transcription profiles of *L. hongkongensis* cultivated with or without malate supplementation, and observed that malate utilization significantly inhibits the use of alternative carbon sources while enhancing respiratory chain as well as central carbon, sulfur, and urease-mediated nitrogen metabolisms. The tight connection among these important metabolic pathways indicates that *L. hongkongensis* is capable of integrating information from different metabolism branches to coordinate the expression of metabolic genes and thereby adapt to environmental changing. Furthermore, we identified that a transcription factor, CRP, is repressed by malate-mediated metabolism while negatively regulating the effect of malate on these central metabolic pathways. Remarkably, CRP also responds to various environmental stresses, influences the expression of other transcription factors, and contributes to the biological fitness of *L. hongkongensis*. The regulatory network and cross-regulation enables the bacteria to make the appropriate metabolic responses and environmental adaptation.

## Introduction

Bacteria have developed sophisticated adaptive mechanisms to resist environmental changes (Bruckner and Titgemeyer, 2002; Deutscher, 2008; Eisenreich et al., 2010; Beisel and Afroz, 2016). This metabolic adaptation to various environmental niches and host is a defining feature for both free-living and pathogenic bacteria (Gorke and Stulke, 2008; de Carvalho et al., 2010; Xiong et al., 2016). Prominent among bacterial adaptive mechanisms to diverse environmental niches are those pertaining to central carbon metabolism which obtain carbon sources to generate energy and produce catabolic intermediates required for the biosynthesis of anabolic macromolecules (Bruckner and Titgemeyer, 2002; Eisenreich et al., 2010). The growth rate of a bacterium is primarily determined by the preferred carbon source and thus directly influences the competitive success of microorganisms (Stulke and Hillen, 1999; Eisenreich et al., 2010; Beisel and Afroz, 2016). Most bacteria can employ various sugars, organic acids, and other organic compounds as carbon sources (Bruckner and Titgemeyer, 2002; Gorke and Stulke, 2008; Meyer et al., 2011; Beisel and Afroz, 2016) and can either co-utilize or favor specific carbon sources that are more easily accessed in order to grow faster (Stulke and Hillen, 1999; Eisenreich et al., 2010). The preference for a certain carbon source over other ones has been termed carbon catabolite repression (CCR) (Stulke and Hillen, 1999; Deutscher, 2008; Gorke and Stulke, 2008), which means the presence of a favored carbon source would inhibit the expression and/or activity of genes responsible for the utilization of secondary substrates. CCR is an environment-dependent mechanism employed by various bacteria which determines the carbon source to use in order to maximize growth and survival in environmental niches (Eisenreich et al., 2010; Soberon-Chavez et al., 2017). CCR has been studied in detail in prokaryotic models including *Escherichia coli* and *Salmonella enterica* serovar Typhimurium (de Crombrugghe et al., 1984; Stulke and Hillen, 1999; Green et al., 2014), and is determined to be modulated by the global transcriptional regulator cyclic AMP (cAMP) receptor protein (CRP), also known as catabolite gene-activator protein (CAP) (Han et al., 2008; Green et al., 2014). In CCR, cAMP production is stimulated by the presence of the preferred carbon source, which activates CRP to bind to the consensus sequence TGTGA-(N6)-TCACA via its helix-turn-helix DNA-binding motif (Zhang and Ebright, 1990; Stulke and Hillen, 1999; Liang et al., 2008). CRP family proteins have diverse functions in different microorganisms and regulate various metabolic pathways (Stulke and Hillen, 1999; Green et al., 2014; Heroven and Dersch, 2014; Yang et al., 2016). In *Deinococcus radiodurans*, the transcription of over 400 target genes is controlled directly or indirectly by CRP (Yang et al., 2016). Furthermore, there are functional interactions between carbon metabolism and other types of metabolism or other metabolic pathways, such as tricarboxylic acid (TCA) cycles, ion utilization, via CRP and related transcription factors (TFs) (Commichau et al., 2006; Shimizu, 2013).

*Laribacter hongkongensis* is a Gram-negative facultative anaerobic bacterium that has been demonstrated to be associated with community-acquired gastroenteritis and stomach and intestinal infection or traveler’s diarrhea (Yuen et al., 2001; Woo et al., 2004), and is suggested to be distributed globally based on the travel records of patients (Woo et al., 2004, 2005; Teng et al., 2005; Kim et al., 2011; Beilfuss et al., 2015; Engsbro et al., 2018). In particular *L. hongkongensis* has been detected in various species of carp, birds, Chinese tiger frogs, as well as reservoirs of drinking water (Lau et al., 2007a, b, 2009; Ni et al., 2011), indicating its remarkably versatile capability to survive in a wide range of environmental niches. As a gastrointestinal tract pathogen (Woo et al., 2004), *L. hongkongensis* has to withstand various hostile environmental niches when transiting from the external environment to the host. The extreme acidic environment of the stomach and the anaerobic condition in the gastrointestinal tract are two of the major environmental stresses encountered during infection (Xiong et al., 2017). We have previously demonstrated the adaptive mechanisms utilized by *L. hongkongensis* to resist acidic and anaerobic conditions via finely regulating arginine metabolism pathways by transcription factors ArgR and FNR (Xiong et al., 2014, 2015, 2017), suggesting the ability to modulate metabolic adaptability in response to hostile stresses encountered in environmental niches or host.

*Lhongkongens hongkongensis* is an asaccharolytic bacterium and is unable to ferment, oxidize, or assimilate most of the common sugars tested as carbon source due to an incomplete set of enzymes for glycolysis and an incomplete phosphotransferase system (Woo et al., 2009). Therefore, *L. hongkongensis* must utilize other available carbon sources for environmental adaptation. Identification of the carbon sources utilized by this bacterium would allow a better understanding of its carbon metabolism under flexible environmental niches and pathogenesis. However, there is limited information on the identity of the carbon sources used by *L. hongkongensis* and related molecular mechanisms. Moreover, as it is an asaccharolytic bacterium, *L. hongkongensis* may obtain its carbon from fatty acids, amino acids, or via the transport of malate by the C4-dicarboxylate family of transporters (Raja and Ghosh, 2014). Among others, malate, a dicarboxylic organic acid, is abundantly found in tissue and in the environment. In accordance, many malate degradation pathways have been discovered among varied microorganisms (Kawai et al., 1996; Augagneur et al., 2007; Kleijn et al., 2010; Landete et al., 2010; Mortera et al., 2012; Paluscio and Caparon, 2015). In our previous analyses according to the Transporter Classification Database (TCDB)^1^, the C4-dicarboxylate uptake C family, belonging to class 2 transporters (Electrochemical potential-driven transporter), is likely to be involved in the transport of malate in *L. hongkongensis* (Groeneveld et al., 2010), which can be utilized by this bacterium as the sole source of carbon in minimal medium (Woo et al., 2009). Moreover, the presence of complete sets of enzymes for gluconeogenesis, the pentose phosphate pathway, and the glyoxylate cycle in the genome of *L. hongkongensis*, further indicates the important role of malate metabolism as an intermediate of these pathways. In this study, we demonstrated that *L. hongkongensis* can preferentially use malate as a carbon source and the global effect of malate utilization on different metabolic pathways was elucidated as well. Given the close relationship of anaerobiosis with the metabolism of this bacterium, as demonstrated in our previous study (Xiong et al., 2017), the transcriptomic data of malate-related genes under anaerobic conditions were also analyzed in parallel. Furthermore, we revealed the involvement of the transcription factor CRP in malate-mediated metabolism. The contribution of CRP for bacterial survival under various environmental stresses and biological fitness was determined in this study as well.

## Materials and Methods

### Bacterial Strains and Conditions for Growth

The bacterial strains and plasmids used in this study are listed in Supplementary Table S2. The wild-type (WT) *L*. *hongkongensis* parental strain HLHK9 was a clinical sample isolated from a patient in Hong Kong (Woo et al., 2009). Unless otherwise noted, bacterial cultures were grown at 37°C either in aerobic (shaking) or anaerobic (static) conditions. Strains of *L*. *hongkongensis* were grown in brain heart infusion (BHI) media (BBL, BD), Lytic/10 Anaerobic/F Medium (BACTEC, BD) or modified minimal medium M63 supplemented with 19 mM potassium nitrate, 1 mM each of vitamin B1 and vitamin B12, with or without malate supplementation as indicated previously (Woo et al., 2009; Xiong et al., 2017). The pH of the medium was adjusted to 7.0 with potassium hydroxide. Strains of *E. coli* were grown in Luria broth (LB) or on LB agar (LBA) plates (Difco, BD) or defined medium. Media were supplemented with the following antibiotics (Sigma-Aldrich) where appropriate: kanamycin (Km, 50 μg/ml), ampicillin (Amp, 100 μg/ml), tetracycline (Tet, 125 ng/ml), streptomycin (Sm, 100 μg/ml), and cefoperazone (Cef, 64 μg/ml). Arginine and malate (Sigma-Aldrich) were prepared as a stock concentrate and filter-sterilized before use. Growth status and bacterial cell density were monitored by measuring the optical density at 600 nm (OD_600_).

### Construction of Non-polar Deletion Mutant Strain HLHK9Δ*crp*

Unmarked, non-polar deletion of *crp* was constructed by homologous recombination using the suicide plasmid pCVD442 as described previously (Xiong et al., 2017). Primers used for the deletion mutagenesis are listed in Supplementary Table S3. Briefly, the in-frame deletion arrangement of *crp* containing its 5′- and 3′-flanking regions was generated using the overlap PCR method and sub-cloned into pCVD442. The resulting plasmid was transferred into HLHK9 by bacterial conjugation from *E*. *coli* SM10 λ pir. The selection of allelic replacement was performed as described previously (Xiong et al., 2015), and the mutant strain was further confirmed by PCR using primers LPW17266/17269 and inner primers (LPW30867/30868) specific for the deleted sequence (Supplementary Table S3). All mutant strains were confirmed by DNA sequencing.

### Growth Kinetics

To determine the growth kinetics of wild-type and mutant strains in modified M63 media, overnight bacterial cultures were diluted 1:50 into fresh M63 broth and further cultured with shaking (250 rpm) at 37°C, with or without malate supplementation (at final concentration of 20 mM) (Woo et al., 2009). To compare the bacterial replicative ability under anaerobic conditions, overnight cultured bacteria were sub-cultured in Lytic/10 Anaerobic/F Medium at 37°C, in the presence or absence of arginine and malate addition as previously described (Xiong et al., 2015). One milliliter of cell suspension was monitored at indicated time points by measuring the absorbance at 600 nm (OD_600_). Experiments were repeated three times in triplicate.

### Response to Different Stress Conditions

*Laribacter hongkongensis* strains were exposed to the following stress conditions before collection of cells for RNA isolation: (i) growth to mid-log phase in modified M63 with or without malate addition, (ii) exposure of mid-log phase cells to acidic conditions (pH 2.0–4.0) and pH 7.0 (PBS) for 60 min, (iii) growth to mid-log phase at 20 and 37°C respectively, and (iv) growth under anaerobic conditions. For each stress condition, bacteria were stabilized first with the RNAprotect bacterial reagent (Qiagen) followed by RNA extraction.

### Extraction of Bacterial RNA

Following treatment with the specific stress condition, bacterial total RNA were extracted using the RNeasy Mini kit (Qiagen) as described by the manufacturer. Extracted RNA was then treated with RNase-free DNase I (Roche) at 37°C for 30 min to decontaminate genomic DNA (Guo et al., 2013). The concentration of purified RNA was quantified using a Nanodrop ND-1000 (NanoDrop Technologies).

### RNA Sequencing and Analysis

The quality and integrity of RNA for RNA sequencing were confirmed by agarose gel electrophoresis (2100 Bioanalyzer, Agilent Technologies, United States). The mRNA was isolated from total RNA samples of three independent replicate biological preparations and pooled together, followed by disrupting fragments as templates for cDNA synthesis. Library construction and sequencing were performed on a BGISEQ-500 platform by Beijing Genomic Institution (^2^ BGI, Shenzhen, China). Clean-tags were mapped to the reference genome of HLHK9^3^ using Bowtie 2 with parameters specifically chosen for RNA-sequencing quantification (Langmead and Salzberg, 2012). The sequenced data was used for calculation of gene expression difference and normalization by the RPKM (Reads Per kb per Million reads) method and RSEM software (Li and Dewey, 2011). The former method normalizes by proportional to the mean length of a transcript, while the latter method is independent of the mean expressed transcript length. Instead, RSEM uses the expectation-maximization algorithm to estimate abundances at the gene level for multiple variables such as library sizes and gene lengths. EBSeq was then used to normalize the RSEM data between different conditions by modeling the differential variability observed in distinct groups to identify differentially expressed genes (DEGs) (Leng et al., 2013). False discovery rate (FDR) control was used in multiple hypothesis testing to correct for *p*-value distribution (Mortazavi et al., 2008) as defined by the bioinformatics service of BGI. The genes with FDR ≤ 0.01 and |log_2_ ratio| ≥ 1.0 were defined as DEGs. KEGG Pathway enrichment analysis in the KEGG Database^4^ was used to identify significantly enriched metabolic or signal transduction pathways in the DEGs (Kanehisa et al., 2008). The heatmap in this study was generated using the MeV software (Howe et al., 2011). The raw sequencing data were submitted to NCBI’s Gene Expression Omnibus (GEO^5^) under accession number GSE 135058.

### Real-Time Quantitative RT-PCR (qRT-PCR)

Real-time qRT-PCR was performed using FastStart DNA Master SYBR Green I Mix reagent kit (Roche) on an ABI7900HT Fast Real Time PCR machine (Applied Biosystems) as described previously (Xiong et al., 2014, 2017). The sequences of the primers used for qRT-PCR are listed in Supplementary Table S2. The mRNA levels of target genes were determined by quantification of cDNA, and the calculated threshold cycle (CT) of the target gene was calculated as 2^(^*^*Ct*^*^Target^^–^ *^*Ct*^*^Reference)^, with normalization to the *rpoB* gene (Xiong et al., 2017). Triplicate assays were performed for each target gene and the data are representative of three independent experiments.

### Construction of *gfp* Reporter Strain and Fluorescence Measurement

The *crp* promoter (P*crp*) was inserted into the suicide vector pVIK165 and transcriptional fused with the promoterless *gfp* gene. The recombinant suicide plasmids were transferred into wild-type HLHK9 by bacterial conjugation, and integrated into the chromosomes of *L. hongkongensis* strains by single homologous recombination as previous described (Xiong et al., 2015). Recombinant plasmid containing P*crp* was generated using primer pairs for PCR and DNA sequencing (Supplementary Table S2). GFP expression was calculated as indicated previously (Xiong et al., 2017). Briefly, bacterial cultures in BHI or modified M63 media with or without malate addition were harvested at mid-log phase, washed twice and resuspended in PBS. GFP signal was measured by a Fusion Universal Microplate Analyzer (VICTOR3; Perkin-Elmer). Relative fluorescence intensity was calculated by subtracting extinction from the PBS background and showed as total fluorescence (FL) units per OD_600_ unit. Each experiment was carried out in triplicate and repeated three times.

### Bacterial Susceptibility of *L. hongkongensis* to Acid pH

Bacterial susceptibility to acidic conditions was evaluated by exposure to a range of acidic pHs from pH 2.0 to 4.0 as previously described (Xiong et al., 2014). Briefly, bacterial cells grown to mid-log phase were incubated in acidic buffer, followed by washing with PBS (pH 7.0). The number of viable cells were determined via serial dilution of each culture and spread on BHA in duplicates. The experiments were repeated three times in triplicate.

### Statistical Analysis

Unless otherwise noted, data generated in the wet experiments were expressed as mean ± standard error of the mean (SEM) from three independent experiments. Statistical analysis for the data collected from wet experiments was carried out using the unpaired Student’s *t*-test with GraphPad Prism 5.0. An asterisk indicates a significant difference (^∗^*p* < 0.05; ^∗∗^*p* < 0.01; ^∗∗∗^*p* < 0.001; ns, not significant).

## Results and Discussion

### Malate Can Be Utilized as a Carbon Source of HLHK9

**FIGURE 1:**
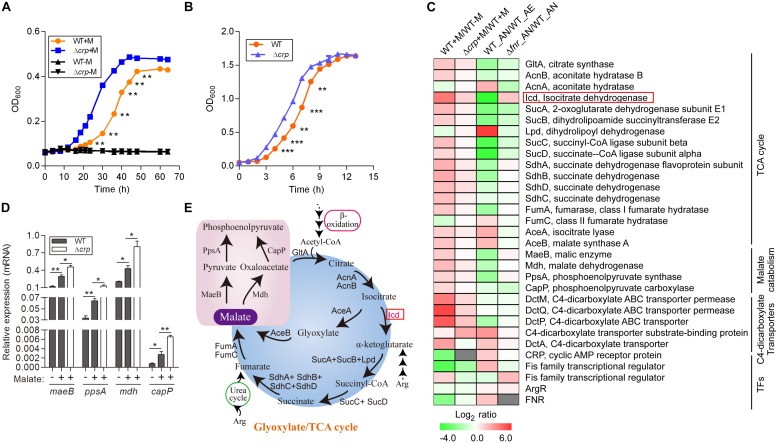
Malate can be utilized as a carbon source by *L. hongkongensis* and elevates the transcript levels of central metabolism. **(A)** Comparison of bacterial growth (WT and Δ*crp* strains) by monitoring OD_600_ over the course of 70 h of aerobic cultures in either modified minimal medium M63 (WT-M; Δ*crp*-M) or M63 supplemented with 0.5% malate (WT + M; Δ*crp* + M). **(B)** Bacterial growth of WT and Δ*crp* strains in BHI broth under aerobic culture condition. Data are presented as means and SEM from three independent experiments. **(C)** Comparison of the transcriptional profiles encoding TCA metabolism and related transcription factors in WT and Δ*crp*, aerobically cultured in modified minimal medium M63 with (WT + M; Δ*crp* + M) or without malate addition (WT-M); as well as in WT and Δ*fnr*, aerobically (WT_AE) and/or anaerobically (WT_ AN; Δ*fnr*_AN) cultured in Lytic/10 Anaerobic/F Medium. Transcript levels were defined by a log_2_ ratio. **(D)** Under the above conditions **(C)**, the transcript levels of four randomly selected genes that encode for central metabolism were determined by qRT-PCR. **(E)** Simplified scheme of TCA/glyoxylate cycles and malate catabolism pathway. Error bars represent means ± SEM of three independent experiments (^∗^*P* < 0.05; ^∗∗^*P* < 0.01; ^∗∗∗^*P* < 0.001).

Carbon sources and related regulatory effects influence a variety of physiological processes including central carbon metabolism, oxidative stress response, virulence, and pathogenesis (Shimizu, 2013; Yang et al., 2016). To determine whether malate can be utilized as a source of carbon for *L. hongkongensis*, the WT HLHK9 was grown either in modified minimal medium M63 alone or in the presence of malate as the sole carbon source. No growth was observed in M63 alone, but bacterial growth yields were dramatically restored in the presence of malate (Figure 1A), indicating that malate can be employed by *L. hongkongensis* as a carbon source. Moreover, in line with its asaccharolytic nature and the results of previous studies, bacterial growth was not restored in the presence of glucose (Yuen et al., 2001 and data not shown).

### Malate Utilization Enhances the Central Carbon Metabolic Pathway

To better understand the malate-mediated metabolism and related regulatory profiles of *L. hongkongensis*, we compared the respective expression profiles of *L. hongkongensis* cells grown in modified M63 media supplemented with or without malate using high-throughput RNA-sequencing. Transcripts that showed significant differences with a FDR ≤ 0.001 and a |log_2_ ratio| ≥ 1.0 were accepted as candidate DEGs. Of the 3,001 genes in the *L. hongkongensis* genome (Woo et al., 2009), the expression of 491 genes was significantly up-regulated and 358 genes were markedly repressed by malate addition (Supplementary Table S1). These 849 malate-regulated genes were classified into six functional classes based on the KEGG pathway analysis (Supplementary Table S1). Among these DEGs, over half (508 of 849) of them were associated with various metabolic pathways. This suggested a close relationship of malate metabolism with other metabolic processes. Of note, 34.7% of the DEGs (295 of 849) were assigned to the ‘function unknown’ class, which encodes hypothetical proteins, conserved hypothetical proteins and proteins with unknown functions (Supplementary Table S1 and Woo et al., 2009).

Among the DEGs associated with central metabolism, those that belonged to the TCA cycle were collectively up-regulated by malate utilization (Figures 1C,D and Supplementary Table S1), suggesting a regulatory effect of malate on central carbon metabolism. Because of the generation of ATP and metabolic intermediates, the TCA cycle is recognized as the most important pathway for cellular energy and metabolism. Specifically, the transcript level of *icd*, encoding isocitrate dehydrogenase, was dramatically augmented by malate-mediated metabolism. Isocitrate dehydrogenase is responsible for the conversion of isocitrate to alpha-ketoglutarate and is the key rate-limiting step of the TCA cycle (Figure 1E). Of note, it was observed that the transcript levels of 9 out of the 16 genes encoding the TCA cycle were dramatically decreased by two-to eight-fold under anaerobic conditions (Figure 1C and Xiong et al., 2017), mainly due to the absence of terminal electron acceptors of oxygen. Furthermore, anaerobiosis dramatically repressed the transcript level of *icd* (Figure 1C), which was observed to be partially mediated by FNR, a global transcriptional regulator of bacteria anaerobiosis demonstrated in our previous study (Xiong et al., 2017 and Figure 1C). The demonstration of the regulatory effects of both malate utilization and anaerobiosis on isocitrate dehydrogenase further confirms its importance and suggests that malate-mediated catabolism and anaerobiosis are closely involved in bacterial adaptation and survival and tightly regulates bacterial central metabolism.

We identified genes encoding a complete set of enzymes for the glyoxylate cycle in *L. hongkongensis*, indicating that there was an economic way for our bacterium to generate anaplerotic and gluconeogenic compounds from acetyl-CoA, thereby utilizing C2 compounds as the sole carbon source (Curreem et al., 2011 and Figure 1E). Acetyl-CoA is directly generated by the oxidation of fatty acids or other lipids (Figure 1E) and the glyoxylate cycle facilitates the anabolism of critical components of bacteria from C2 units by producing C4 intermediates that function as biosynthetic precursors (Munoz-Elias and McKinney, 2005). In accordance with this, the transcript levels of genes responsible for the glyoxylate cycle, such as *aceA*, which encodes for isocitrate lyase, and *aceB*, which encodes for malate synthase, were significantly enhanced by sixfold by malate utilization (Figure 1C), indicating the involvement of the glyoxylate cycle in the central carbon metabolism of *L. hongkongensis*. Of note, accumulating evidence has demonstrated that isocitrate lyase, the enzyme responsible for the initial step of the glyoxylate cycle, is involved in the virulence of various pathogenic bacteria (McKinney et al., 2000; Vereecke et al., 2002; Munoz-Elias and McKinney, 2005; Gouzy et al., 2014) as well as fungal pathogens (Lorenz and Fink, 2001; Wang et al., 2003). It would be interesting to further investigate the pathogenic function of isocitrate lyase the life cycle of *L. hongkongensis*.

### Malate Represses Genes for Utilization of Alternative Carbon Sources

As *L. hongkongensis* is an asaccharolytic bacterium that is unable to ferment, oxidize, or assimilate most common sugars tested as carbon source, it must utilize other available carbon sources, such as malate, as identified in the present study. Furthermore, based on the whole genomic analysis, *L. hongkongensis* contains genes encoding enzymes for various pathways responsible for the usage of alternative carbon sources, such as the biosynthesis of different universal amino acids and selenocysteine, anabolism and β-oxidation of saturated fatty acids (Woo et al., 2009; Curreem et al., 2011), suggesting that amino acid catabolism and β-oxidation of fatty acids may also be utilized as carbon or energy sources.

**FIGURE 2:**
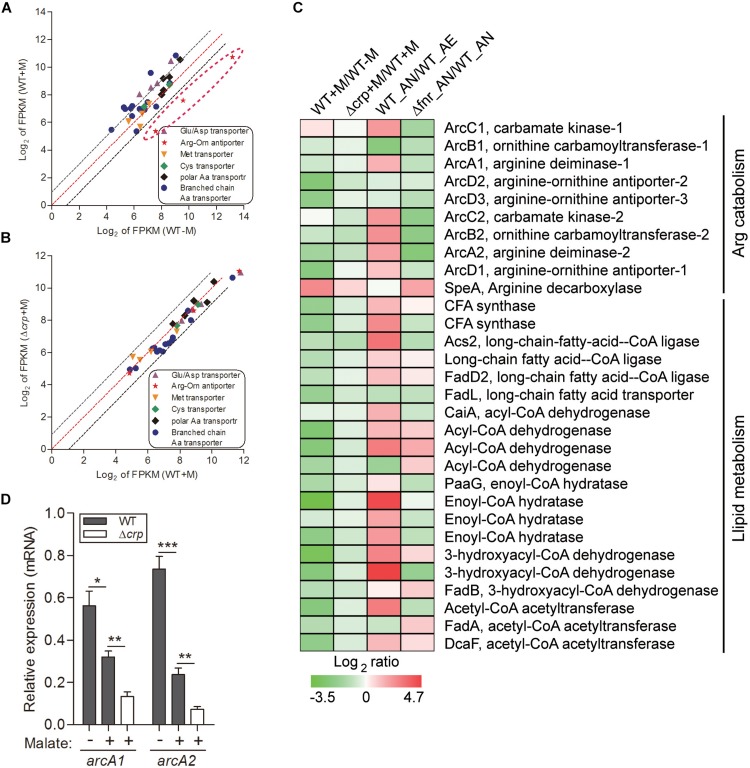
Malate utilization represses alternative carbon sources of arginine catabolism and fatty acid β-oxidation, which is partially mediated by CRP. The relative expression of genes encoding various amino acid transporters were compared in WT strain under malate present (WT + M) and absent (WT-M) conditions **(A)**, and between WT and Δ*crp* strains with malate supplementation (WT + M; Δ*crp* + M) **(B)**. Each dot is representative of a single gene. The dashed lines represent a twofold over-expression (black, upper line), equal-expression (red, middle line) and –2-fold expression (black, lower line) of ratio of axis Y/X defined by log_2_ of FPKM. **(C)** Comparison of the transcriptional profiles encoding *arc* operons and fatty acid β-oxidation in WT and Δ*crp*, aerobically cultured in modified minimal medium M63 with (WT + M; Δ*crp* + M) or without malate addition (WT-M); as well as in WT and Δ*fnr*, aerobically (WT_AE) and/or anaerobically (WT_ AN; Δ*fnr*_AN) cultured in Lytic/10 Anaerobic/F Medium. **(D)** Under the above conditions **(C)**, the transcript levels of two randomly selected genes that encode for arginine catabolism were determined by qRT-PCR. Error bars represent means ± SEM of three independent experiments (^∗^*P* < 0.05; ^∗∗^*P* < 0.01; ^∗∗∗^*P* < 0.001).

Employment of single amino acids as the bacterial carbon and nitrogen source is common and widely distributed over many genera. The metabolic products from these reactions can further participate in other metabolic pathways as intermediates, such as the TCA cycle and gluconeogenesis pathway. We analyzed the transcription profiling of various amino acid transporters and found that only the transporters of Arg/Orn were markedly repressed by the presence of malate (Figure 2A). Arginine participates in the TCA cycle via the urea cycle or acts as an intermediate of α-ketoglutarate (Figure 1E). These results suggest that the use of arginine as an alternative carbon source is inhibited by the presence of malate. Of note, we have previously demonstrated that arginine catabolism mediated by the *arc* operons plays a vital role as carbon/nitrogen/energy source for bacterial adaptation under anaerobic conditions (Xiong et al., 2017). The transcriptomic data of malate-related pathways under anaerobiosis was thus also analyzed in parallel. Remarkably, the expressions of genes encoding *arc* operons were observed to be collectively reduced by the presence of malate (Figure 2C). This is consistent with our growth assay that arginine addition did not significantly affect bacterial growth (data not shown). However, the transcript level of s*peA*, which encodes for arginine decarboxylase, was significantly increased by malate supplementation (Figure 2C), indicating that SpeA was exploited by this bacterium to further reduce arginine concentration under malate utilization. In addition, two *arc* operon-encoding genes were randomly selected for the assessment of their mRNA levels to confirm the transcriptomic data, using qRT-PCR (Figure 2D).

Besides amino acid metabolism, fatty acids respiration has also been demonstrated to be exploited as an important source of carbon by some pathogenic bacteria. For example, extensive studies have shown that during infection, mycobacteria predominantly use fatty acids, instead of carbohydrates, as carbon substrates (Munoz-Elias and McKinney, 2005). Consistently, genes encoding enzymes responsible for mycobacterial fatty acid metabolism are up-regulated in macrophages and mice (Munoz-Elias and McKinney, 2005). Fatty acids catabolism is primarily mediated by β-oxidation, where the products of acetyl-CoA are utilized for the TCA cycle, and NADH and FADH_2_ are exploited for ATP synthesis (Boshoff and Barry, 2005 and Figure 1E). In line with this, we identified the genes encoding all of enzymes responsible for the β-oxidation of saturated fatty acids, but not unsaturated fatty acids, in the genome of *L. hongkongensis* (Curreem et al., 2011), indicating that these fatty acids may be employed by *L. hongkongensis* as a carbon/energy source. Our transcriptomic data showed that transcript levels of genes encoding for fatty acid β-oxidation were dramatically decreased by malate-mediated metabolism (Figure 2C). Moreover, the expression of two copies of cyclopropane-fatty-acyl-phospholipid synthases (CFA synthase) was also repressed by malate utilization (Figure 2C), suggesting that *L. hongkongensis* can employ cyclopropane fatty acid for metabolic purposes. In *E. coli*, cyclopropane fatty acids found in the bacterial membrane contributes to resist acidic conditions, while these fatty acids are observed to be associated with virulence and pathogenesis in *Mycobacterium tuberculosis* (Chang and Cronan, 1999; Barkan et al., 2009). Taken together, we have demonstrated in the present study that malate-mediated metabolism represses alternative carbon sources including amino acids and fatty acids, suggesting that malate is a preferred carbon source of *L. hongkongensis*.

### Urease-Mediated Nitrogen Metabolism Is Tightly Connected With Malate-Mediated Carbon Metabolism and Anaerobic Adaptation

**FIGURE 3:**
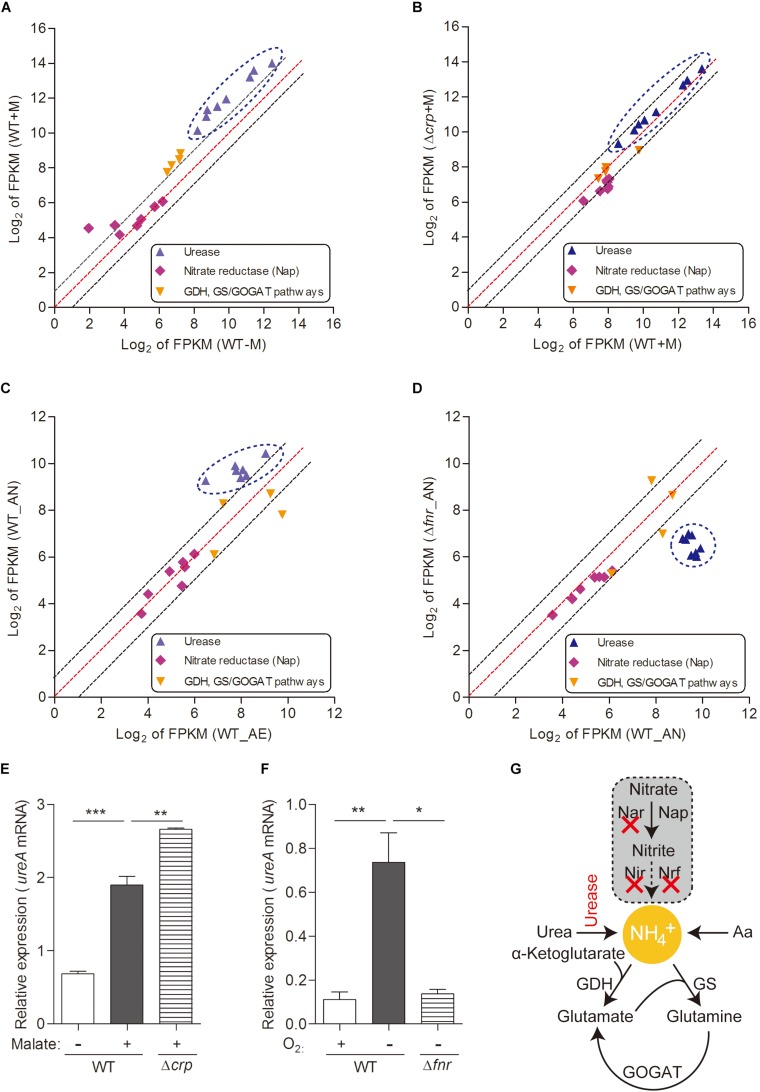
Urease-mediated nitrogen metabolism of *L. hongkongensis* is significantly enhanced by malate addition and anaerobisis, which is partially regulated by CRP and FNR respectively. Comparison of the relative expression of genes encoding nitrogen metabolism in WT and Δ*crp*, aerobically cultured in modified minimal medium M63 with (WT + M; Δ*crp* + M) or without malate addition (WT-M) **(A,B),** as well as in WT and Δ*fnr*, aerobically (WT_AE) and/or anaerobically (WT_ AN; Δ*fnr*_AN) cultured in Lytic/10 Anaerobic/F Medium **(C,D)** by transcriptomic analyses. The genes from the same pathway are shown in the same color and each dot is representative of a single gene. The dashed lines represent a twofold over-expression (black, upper line), equal-expression (red, middle line) and –2-fold expression (black, lower line) of ratio of axis Y/X defined by log_2_ of FPKM. Under the above conditions **(A–D)**, the transcript levels of genes encoding for urease-mediated nitrogen metabolism were determined by qRT-PCR **(E,F)**. **(G)** Simplified organization of nitrogen metabolism in *L. hongkongensis*. Error bars represent means ± SEM of three independent experiments (^∗^*P* < 0.05; ^∗∗^*P* < 0.01; ^∗∗∗^*P* < 0.001).

Amino acids and nucleic acids are vital biomolecules in organisms and their breakdown via nitrogen metabolism is an essential pathway (Shimizu, 2013; Gouzy et al., 2014), which has tight connections with carbon metabolism (Commichau et al., 2006). Ammonium is an essential element of central nitrogen metabolism in bacteria (Gouzy et al., 2014), and can be assimilated by two general pathways distributed in various microorganisms: the low-affinity pathway, which is catalyzed by glutamate dehydrogenase (GDH), with the production of glutamate; and the high-affinity pathway, which uses glutamine synthetase (GS) and glutamine oxoglutarate aminotransferase (GOGAT), to convert glutamate to glutamine, with the incorporation of ammonia (Figure 3G).

Bacteria have adapted to exploit various sources of nitrogen, including inorganic compounds such as ammonia, which is the preferred source of nitrogen for most bacteria (Gouzy et al., 2014) which can be provided by urea assimilation and/or nitrate utilization (Figure 3G); and organic complex compounds such as amino acids (Curreem et al., 2011; Gouzy et al., 2014). In *L. hongkongensis*, a complete urease gene cluster is present (Woo et al., 2009). Since the final product of the urease pathway is a nitrogen source (urea), it can be exploited by *L. hongkongensis* as a strategy for nitrogen source adaptation. To examine this hypothesis, we tested the transcript levels of genes encoding the urease cassette and observed the significant increase of these genes by malate-mediated carbon metabolism (Figure 3A). Moreover, the transcript levels of genes encoding the urease cassette were also significantly increased under anaerobiosis (Figure 3C), indicating that urease-mediated nitrogen metabolism is also vital for bacterial adaptation and survival under anaerobic conditions. This enhancement is also mediated by FNR, as confirmed by our results in the current study (Figure 3D). The regulatory effect of both of malate addition and anaerobiosis on urease cassette was further demonstrated using qRT-PCR (Figures 3E,F). Nitrate is another common form of inorganic compounds that can be exploited as a nitrogen source. However, the genome of *L. hongkongensis* only contains the periplasmic (Nap-type) nitrate reductase; other critical nitrate reductases that reduce nitrate into building blocks are absent (Curreem et al., 2011 and Figure 3G). Moreover, we did not observe significant changes of the transcript level of Nap nitrate reductase under the examined conditions (Figures 3A–D). This further suggests that *L. hongkongensis* is highly reliant on urease-mediated urea catabolism to serve as a nitrogen source.

### Malate-Mediated Metabolism Enhances Sulfur Metabolism

**FIGURE 4:**
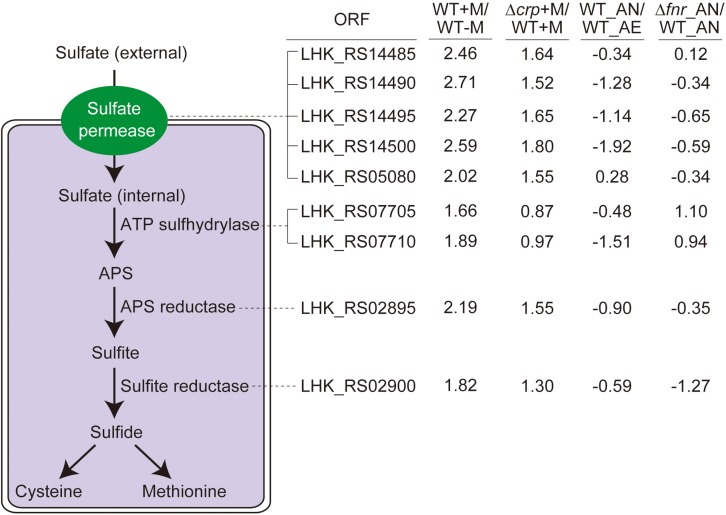
The sulfate assimilation pathway is enhanced by malate utilization and negatively affected by CRP, but not significantly influenced by anaerobiosis. The sulfate assimilation pathway converts inorganic sulfate to sulfide and enter L-cysteine and L-methionine biosynthetic pathways, with the corresponding ORF associated with each enzymatic step. The transcriptomic data were collected from the same treatment as indicated in Figure 3. The log_2_ ratio of different treatments is shown alongside the ORF.

As an important component in various metabolites, sulfur is not only distributed in amino acids, i.e., methionine and cysteine, but also involved in some coenzymes (Curreem et al., 2011). The genome of *L. hongkongensis* consists of genes encoding the whole set of enzymes responsible for the sulfate assimilation pathway (sulfate permease, ATP sulfhydrylase, ATP reductase, and sulfite reductase), suggesting the possibility that sulfur can be utilized to generate sulfide in *L. hongkongensis* and contribute to the anabolism of L-cysteine and L-methionine. We determined the expression of these genes in transcriptomic analyses and observed that they were collectively elevated by malate metabolism (Figure 4), indicating that malate metabolism was involved in regulating the sulfate assimilation pathway. On the other hand, anaerobiosis did not significantly affect sulfur metabolism (Figure 4).

### Malate-Mediated Metabolism Enhances the Respiratory Chain

**FIGURE 5:**
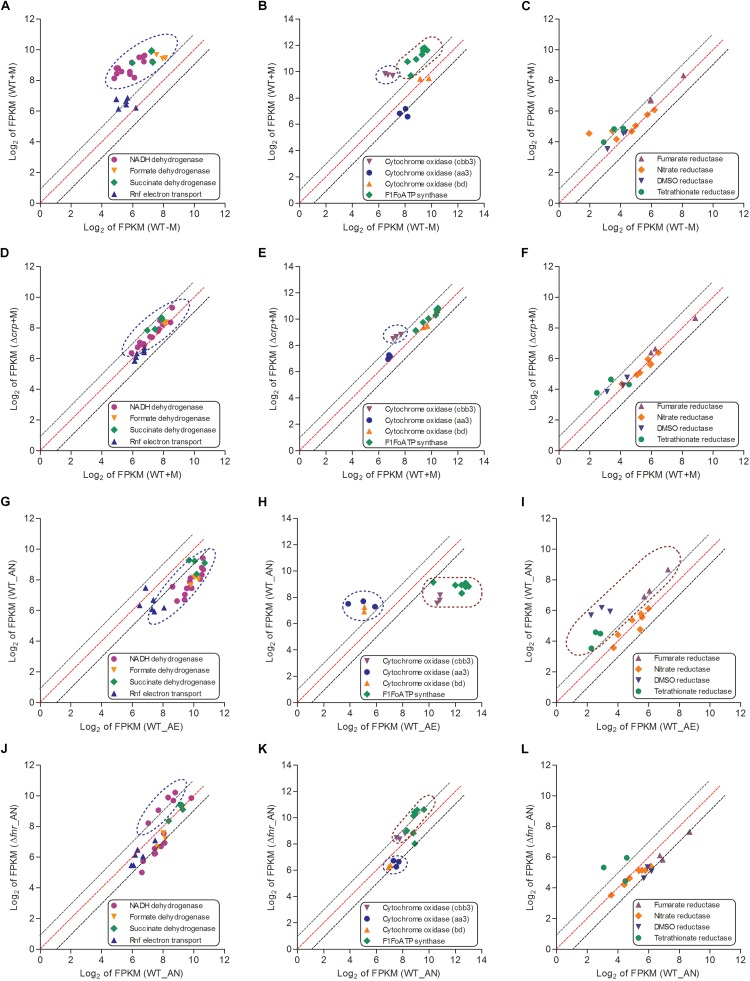
The respiratory chains (including electron donors, electron acceptors for aerobic respiration and electron acceptors for anaerobic respiration) of *L. hongkongensis* were enhanced by malate addition while reduced under anaerobic condition, which were partial mediated by CRP and FNR respectively. The relative expression of genes encoding respiratory chains were compared in WT strain under malate present (WT + M) and absent conditions (WT-M) **(A–C)**, and between WT and Δ*crp* strains with malate supplementation (WT + M; Δ*crp* + M) **(D–F)**. The transcriptional profiles of these genes were also compared in WT strain under aerobic (WT_AE) and anaerobic conditions (WT_AN) **(G–I)**, and between WT and Δ*fnr* strains under anaerobic conditions (WT_ AN; Δ*fnr*_AN) **(J–L)**. Each dot is representative of a single gene. The dashed lines represent a twofold over-expression (black, upper line), equal-expression (red, middle line) and –2-fold expression (black, lower line) of ratio of axis Y/X defined by log_2_ of FPKM.

An increase of TCA cycle activity under aerobic conditions enhances NADH production and thereby activates respiration (Shimizu, 2013). The major role of the bacterial respiratory chain is transport of electrons from electron donors to acceptors, and the generation of proton gradient during this process contributes to ATP biosynthesis via F_1_F_o_ ATP synthase, a gene which is identified in the genome of *L. hongkongensis* (Curreem et al., 2011). Bacteria can use diverse substrates as electron donors, such as NADH, formate and succinate, in which electrons enter the electron transport chain via related dehydrogenases, which are all encoded in *L. hongkongensis*. This raised the possibility that these dehydrogenases may be involved in the metabolism of our bacterium. Accordingly, we analyzed the transcript levels of genes encoding for these dehydrogenases and found that most of them were significantly enhanced by malate supplementation (Figure 5A). However, when grown in anaerobic environments, bacteria cannot use these dehydrogenases as electron donors to produce ATP and the expression levels of most dehydrogenases were dramatically reduced accordingly (Figure 5G), which was partially mediated by FNR (Figure 5J).

When a bacterium is grown in aerobic conditions, the enzyme oxidase functions for the reduction of the terminal electron acceptor (O_2_). We identified genes encoding three terminal cytochrome oxidases in the genome of *L. hongkongensis*: two haem-copper oxidases (type aa3 oxidase and type cbb3 oxidase) and a quinol oxidase (type bd oxidase). The type aa3 oxidase functions for respiration under aerobic conditions, while the type cbb3 and type bd oxidases functions under reduced oxygen tension conditions (Curreem et al., 2011). Among the three cytochrome oxidases, only the transcript level of cbb3 oxidase was significantly elevated by malate addition (Figure 5B). In contrast, the transcription profiling of cbb3 oxidase was dramatically repressed under anaerobic conditions (Figures 5H,K). Of note, type cbb3 oxidase is the most ancient oxidase among the three cytochrome oxidases and is found in almost all proteobacteria except for the anaerobic δ-proteobacteria (Myllykallio and Liebl, 2000). The expression of F_1_F_o_ ATP synthase was found to also exhibit a similar regulatory trend as that of cbb3 oxidase (Figures 5B,E).

When a bacterium is grown in anaerobic conditions, the enzyme reductase contributes to the reduction of the terminal electron acceptor. Genes encoding for various reductases were detected in the *L. hongkongensis* genome, including dimethylsulfoxide (DMSO) reductase, nitrate reductase, fumarate reductase, and tetrathionate reductase (Curreem et al., 2011), which raised the possibility that our bacterium may employ them as alternative electron acceptors (DMSO, nitrate, fumarate, and tetrathionate, respectively) to oxygen under anaerobic conditions. Transcriptomic data showed that the expression of fumarate reductase, DMSO reductase and tetrathionate reductase were not significantly affected by malate supplementation (Figures 5C,F). On the other hand, the transcript level of these reductases were significantly enhanced under anaerobic conditions (Figure 5I), and were partially influenced by FNR (Figure 5L). Remarkably, DMSO is abundant in aquatic environments, so the involvement of DMSO reductase in anaerobic adaptation is in line with the bacterial living niches as found in the freshwater environment and in fishes (Lau et al., 2007b, 2009). Notably, among members of the family *Neisseriaceae* with complete bacterial genome sequences available, the *ttr* operon, which encodes the active tetrathionate reduction system and is responsible for tetrathionate respiration, is detected only in the genome of *L. hongkongensis* (LHK_RS06770-LHK_RS06780). The use of tetrathionate reductase as an electron acceptor indicates the importance of tetrathionate utilization for anaerobic adaptation, which was observed in several genera of *Enterobacteriaceae* including *Citrobacter*, *Salmonella*, *Yersinia*, and *Proteus* (Barrett and Clark, 1987). Furthermore, *S*. *enterica* serovar Typhimurium was found to employ the tetrathionate respiration to compete with the microbiota in the gut so as to promote its growth in the gut during inflammation (Winter et al., 2010). Specifically, the reactive oxygen species generated by inflammation react with thiosulfate to produce tetrathionate sources (Winter et al., 2010). Given the association of *L. hongkongensis* with diarrhea, the presence of genes encoding the complete tetrathionate reduction system indicate that our bacterium may exploit the similar electron acceptor (tetrathionate) strategy for generating a growth advantage during infection.

### Identification of CRP Regulator Repressed by Malate-Mediated Metabolism

Among the DEGs regulated by malate utilization, a transcriptional regulator named CRP (encoded by gene LHK_RS10955) was observed to be dramatically repressed in the presence of malate (Figure 1C). CRP is a global transcriptional regulator that is widely distributed among bacteria. It mediates CCR by binding at specific consensus sequences (Stulke and Hillen, 1999; Liang et al., 2008). Using the protein domain analysis of PROSITE web-server^6^ (Sigrist et al., 2010), we identified a typical conserved helix-turn-helix (HTH) DNA binding motif in the CRP of *L. hongkongensis* (data not shown), implying the CRP may exert similar regulatory function as demonstrated in other bacterial species.

**FIGURE 6:**
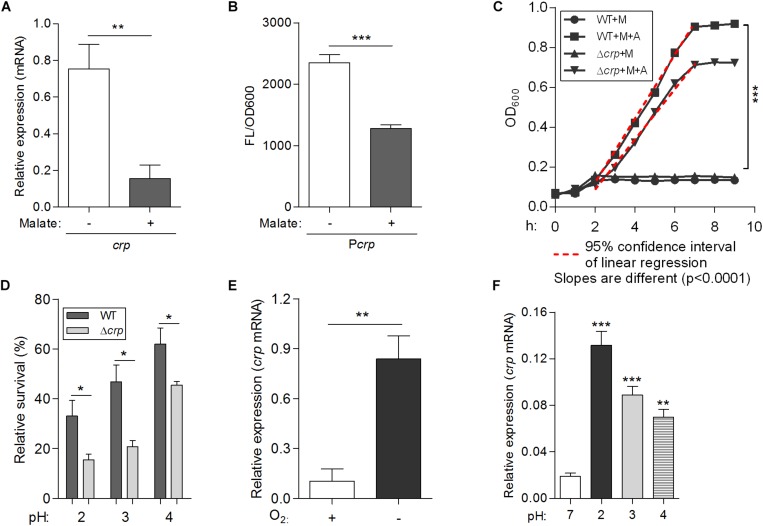
A newly identified transcriptional regulator CRP, negatively regulates malate-mediated metabolism and contributes to the biological fitness of *L. hongkongensis*. Comparison of the mRNA levels of *crp* **(A)** and promoter activities of P*crp* **(B)** in wild-type HLHK9, cultivated in modified minimal media M63, in the absence or presence of malate. The P*crp* was transcriptionally fused with *gfp* gene as described in the Experimental Procedures, and promoter activity was monitored by comparing GFP fluorescence/OD_600_. **(C)** Growth curves of HLHK9 versus derivative mutant strain Δ*crp* grown under anaerobic conditions, in the absence or presence of malate [M] and arginine [A]. The optical density at 600 nm (OD_600_) was measured hourly over 10 h. **(D)** Survival of HLHK9 and derivative mutant Δ*crp* under acidic conditions. Survivors were enumerated by plating serial dilutions on BHA plates. Comparison of the mRNA levels of the gene encoding for CRP in *L. hongkongensis*, under aerobic or anaerobic conditions **(E)**, and normal (pH 7.0) or acidic conditions (pH 2.0–4.0) **(F)**. Error bars represent means ± SEM of three independent experiments. Asterisks indicate a significant difference (^∗^*P* < 0.05; ^∗∗^*P* < 0.01; ^∗∗∗^*P* < 0.001).

To confirm the inhibitory effect of malate addition on *crp* expression in *L. hongkongensis*, the expression level of *crp* was measured in the WT strain in the absence and presence of malate, using qRT-PCR. Consistent with the transcriptomic data (Figure 1C), the transcriptional level of *crp* was dramatically decreased in the presence of malate (Figure 6A). The effect of malate on the promoter activity of the *crp* (P*crp*) was further evaluated using a plasmid-based *gfp* gene reporter system. Reporter strains were constructed in WT HLHK9, and contained chromosomal copies of the *gfp* reporter gene immediately downstream of the P*crp*. Reporter activity was then observed by measuring fluorescence produced by the green fluorescent protein (GFP) with or without malate supplement. Malate addition was observed to significantly reduce the relative fluorescence intensity levels (Figure 6B), indicating a substantial reduction of P*crp* activity. These results demonstrate the negative regulatory effect of malate on *crp* expression in *L. hongkongensis*.

### CRP Negatively Regulates Malate Utilization and Malate-Mediated Metabolism in *L. hongkongensis*

To explore the role of *crp* in malate-mediated metabolism of *L. hongkongensis*, an in-frame deletion mutant HLHK9Δ*crp* was constructed, and the growth profile of the mutant was compared to that of the HLHK9 WT strain. As shown in Figure 1A, a comparable reduction in the rate of growth was detected in Δ*crp* and the WT strain when cultured in modified medium M63 without malate. However, growth with malate supplementation resulted in a faster growth rate for Δ*crp* mutant as compared to that of the WT strain. The enhanced growth profile of Δ*crp* was also observed in rich media (BHI broth) when compared with that of the WT strain (Figure 1B). Our results suggested that the CRP regulator is involved in the negative regulation of malate metabolism in *L. hongkongensis* as a negative feedback loop.

To comprehensively study the regulatory effect of CRP on malate-mediated metabolism in *L. hongkongensis*, the transcription profile of HLHK9Δ*crp* was globally evaluated by transcriptomic analyses and was compared with that of WT (Supplementary Table S1). Since CRP expression was already dramatically repressed by the presence of malate in the WT strain, there was limited capacity for further reduction of CRP expression in the Δ*crp* mutant, hence the effect of *crp*-deficiency on the expression of regulated genes was greatly compromised and very few DEGs were included in the transcriptomic data using the same cutoff mentioned above (Figures 1C, 2B and Supplementary Table S1). However, when we further analyzed the whole transcriptomic data (Supplementary Table S4), it was clearly observed that CRP had a regulatory effect on malate-mediated metabolism in *L. hongkongensis*. Firstly, the transcript levels of genes encoding the central metabolic pathway that were enhanced by malate utilization, such as *aceA*, *icd* and *mdh*, were further elevated by *crp* deletion (Figure 1C), indicating the negatively regulatory effect of CRP on malate-mediated TCA cycles. This negative effect was further confirmed by qRT-PCR (Figure 1D). Secondly, the repressive influence of malate on alternative carbon sources, arginine catabolism (such as *arc* operons) and fatty acid beta-oxidation (such as CFA synthase), was also partially attenuated by CRP, as elucidated by both transcriptomic analysis and qRT-PCR (Figures 2C,D). Thirdly, deletion of *crp* further collectively elevated the expression of genes encoding the urease cassette (such as *ureA*) (Figures 3B,E), sulfur metabolism (such as sulfate permease) (Figure 4) and respiratory chain (such as *cbb3*) (Figures 5A–F). Taken together, we demonstrated the negative regulatory role of CRP over malate-mediated metabolism in *L. hongkongensis*.

### CRP Responds to Various Environmental Stresses and Contributes to Biological Fitness of *L. hongkongensis*

To determine the contribution of CRP to bacterial biological fitness, we further compared the adaptive capability of WT and Δ*crp* mutant strains under stress conditions. In our previous study, we demonstrated that HLHK9 has versatile abilities to adapt to various hostile environmental niches. Among others, the extreme acidic environment of the stomach and the limited supply of oxygen present within the gastrointestinal tract are two major environmental stresses encountered during infection (Xiong et al., 2015, 2017). The adaptation of *L. hongkongensis* to these environmental stresses was studied in the present study.

The transcript level of *crp* was observed to be significantly increased under anaerobiosis (Figure 1C), suggesting the involvement of CRP in anaerobic adaptation. First, we evaluated the growth kinetics of WT HLHK9 and derivative mutant Δ*crp* strain under anaerobic conditions, in the absence or presence of malate and arginine supplement. Consistent with our previous data, the wild-type strain HLHK9 replicated slowly over time under anaerobic conditions without arginine addition (Xiong et al., 2017), and the Δ*crp* mutant exhibited a similar growth defect in comparison with that of WT (Figure 6C). In the presence of arginine and malate, the growth of WT was completely restored under anoxic conditions, while deletion of *crp* markedly compromised bacterial growth (Figure 6C). HLHK9 and Δ*crp* mutant strain were further exposed to a range of acidic pHs (from pH 2.0 to 4.0) in the presence of 25 mM of L-arginine. In accordance with our previous study (Xiong et al., 2014), we observed good survival of WT *L. hongkongensis* HLHK9 under the tested pHs. However, deletion of *crp* rendered the bacteria less resistant to acidic exposure and significantly decreased its survival (Figure 6D). Consistently, the expression levels of *crp* was markedly increased under anaerobic conditions (Figure 6E) and acidic pHs (Figure 6F). Furthermore, we assessed the mRNA level of *crp* under growth temperatures of 20 and 37°C and did not observe any significant differences in growth rate (data not shown), which is in line with our previous transcriptomic data (Kong et al., 2017). These findings emphasize the important contributions of the transcription factor CRP for the biological fitness of *L. hongkongensis*.

## Conclusion

**FIGURE 7:**
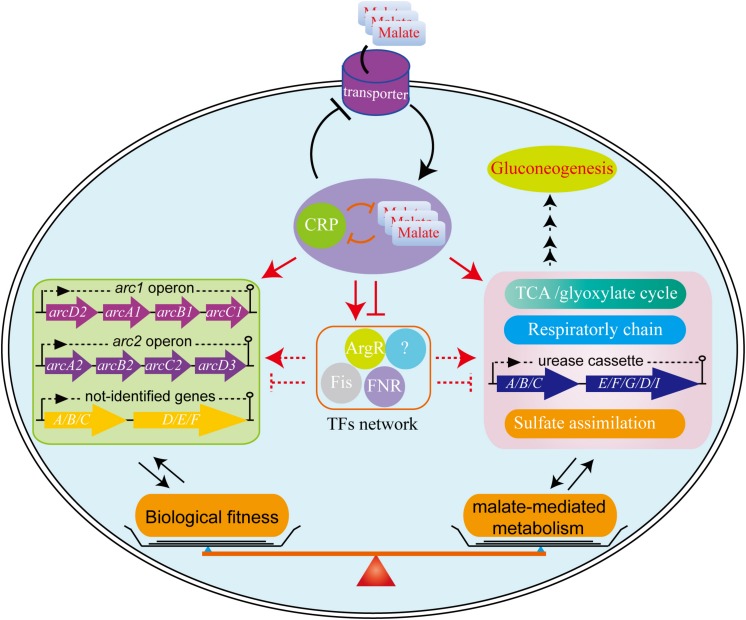
The proposed model for biological fitness mediated by malate in *L. hongkongensis*. Malate can be utilized as a sole carbon source and enhance the central metabolism, urease-mediated nitrogen metabolism, sulfate assimilation and respiratory metabolism. On the other hand, this process is precisely regulated by the transcription factor CRP, which can negatively balance this process by regulating other transcription factors (TFs) or target genes directly or indirectly, to form transcriptional regulatory networks and delicate feedback loop, thereby finely monitoring bacterial adaptation to various stresses.

We have completed an extensive analysis of the regulatory mechanisms associated with malate utilization as a source of carbon on the central metabolic pathway of *L. hongkongensis*. Our data clearly demonstrates that malate in *L. hongkongensis* represses the utilization of alternative carbon sources, suggesting an important role of malate in the physiology of our bacterium as a preferred carbon source. The utilization of malate by *L. hongkongensis* has also led to significant enhancement of respiratory chain activity as well as central carbon, sulfur and urease-mediated nitrogen metabolisms. We further identified the transcription factor of CRP as a negative regulator of malate-mediated metabolism by forming transcriptional regulatory networks via regulating other transcription factors or target genes, as summarized in Figure 7. Remarkably, CRP also responds to various environmental stresses and contributes to the biological fitness of *L. hongkongensis*, which have been demonstrated to be tightly linked to bacterial virulence and pathogenicity. We also demonstrated the close relationship of malate-mediated catabolism with anaerobiosis for bacterial adaptation. Our new findings provide a comprehensive understanding of carbon/nitrogen metabolism of *L. hongkongensis* and emphasize the outstanding importance of metabolic adaptation for coordinating biological fitness, and possibly, the pathogenesis of our bacterium.

## Data Availability

All datasets generated for this study are included in the manuscript and/or the Supplementary Files.

## Author Contributions

LX and PW conceived the study. LX and EC performed the experiments. LX wrote the manuscript. EC, JT, SL, SKPL, and PW discussed the results and revised the manuscript. All authors read and approved the final manuscript.

## Conflict of Interest Statement

PW has provided scientific advisory/laboratory services for Gilead Sciences, Inc., International Health Management Associates, Inc./Pfizer, Inc., and Merck & Co., Inc. The remaining authors declare that the research was conducted in the absence of any commercial or financial relationships that could be construed as a potential conflict of interest.

## Footnotes

1 http://www.tcdb.org/

2 www.genomics.org.cn

3 https://www.ncbi.nlm.nih.gov/nuccore/NC_012559.1

4 http://www.genome.jp/kegg/

5 http://www.ncbi.nlm.nih.gov/geo/

6 http://prosite.expasy.org/prosite.html

## References

[B1] Augagneur Y. Ritt J. F. Linares D. M. Remize F. Tourdot-Marechal R. Garmyn D. (2007). Dual effect of organic acids as a function of external pH in *Oenococcus oeni*. *Arch. Microbiol.* 188 147–157. 10.1007/s00203-007-0230-230 17406856

[B2] Barkan D. Liu Z. Sacchettini J. C. Glickman M. S. (2009). Mycolic acid cyclopropanation is essential for viability, drug resistance, and cell wall integrity of *Mycobacterium tuberculosis*. *Chem. Biol.* 16 499–509. 10.1016/j.chembiol.2009.04.001 19477414 PMC2731493

[B3] Barrett E. L. Clark M. A. (1987). Tetrathionate reduction and production of hydrogen sulfide from thiosulfate. *Microbiol. Rev.* 51 192–205. 3299028 10.1128/mr.51.2.192-205.1987 PMC373103

[B4] Beilfuss H. A. Quig D. Block M. A. Schreckenberger P. C. (2015). Definitive identification of laribacter hongkongensis acquired in the united states. *J. Clin. Microbiol.* 53 2385–2388. 10.1128/jcm.00539-515 25948608 PMC4473238

[B5] Beisel C. L. Afroz T. (2016). Rethinking the hierarchy of sugar utilization in bacteria. *J. Bacteriol.* 198 374–376. 10.1128/jb.00890-815 26574509 PMC4719460

[B6] Boshoff H. I. Barry C. E. (2005). A low-carb diet for a high-octane pathogen. *Nat. Med.* 11 599–600. 10.1038/nm0605-599 15937469

[B7] Bruckner R. Titgemeyer F. (2002). Carbon catabolite repression in bacteria: choice of the carbon source and autoregulatory limitation of sugar utilization. *FEMS Microbiol. Lett.* 209 141–148. 10.1111/j.1574-6968.2002.tb11123.x 12007797

[B8] Chang Y. Y. Cronan J. E. Jr. (1999). Membrane cyclopropane fatty acid content is a major factor in acid resistance of *Escherichia coli*. *Mol. Microbiol.* 33 249–259. 10.1046/j.1365-2958.1999.01456.x 10411742

[B9] Commichau F. M. Forchhammer K. Stulke J. (2006). Regulatory links between carbon and nitrogen metabolism. *Curr. Opin. Microbiol.* 9 167–172. 10.1016/j.mib.2006.01.001 16458044

[B10] Curreem S. O. Teng J. L. Tse H. Yuen K. Y. Lau S. K. Woo P. C. (2011). General metabolism of Laribacter hongkongensis: a genome-wide analysis. *Cell Biosci.* 1:16. 10.1186/2045-3701-1-16 21711917 PMC3125206

[B11] de Carvalho L. P. Fischer S. M. Marrero J. Nathan C. Ehrt S. Rhee K. Y. (2010). Metabolomics of Mycobacterium tuberculosis reveals compartmentalized co-catabolism of carbon substrates. *Chem. Biol.* 17 1122–1131. 10.1016/j.chembiol.2010.08.009 21035735

[B12] de Crombrugghe B. Busby S. Buc H. (1984). Cyclic AMP receptor protein: role in transcription activation. *Science* 224 831–838. 10.1126/science.6372090 6372090

[B13] Deutscher J. (2008). The mechanisms of carbon catabolite repression in bacteria. *Curr. Opin. Microbiol.* 11 87–93. 10.1016/j.mib.2008.02.007 18359269

[B14] Eisenreich W. Dandekar T. Heesemann J. Goebel W. (2010). Carbon metabolism of intracellular bacterial pathogens and possible links to virulence. *Nat. Rev. Microbiol.* 8 401–412. 10.1038/nrmicro2351 20453875

[B15] Engsbro A. L. Nielsen K. L. Hornum M. Andersen L. P. (2018). Laribacter hongkongensis: clinical presentation, epidemiology and treatment. a review of the literature and report of the first case in denmark. *Infect. Dis.* 50 417–422. 10.1080/23744235.2017.1419373 29272955

[B16] Gorke B. Stulke J. (2008). Carbon catabolite repression in bacteria: many ways to make the most out of nutrients. *Nat. Rev. Microbiol.* 6 613–624. 10.1038/nrmicro1932 18628769

[B17] Gouzy A. Poquet Y. Neyrolles O. (2014). Nitrogen metabolism in *Mycobacterium tuberculosis* physiology and virulence. *Nat. Rev. Microbiol.* 12 729–737. 10.1038/nrmicro3349 25244084

[B18] Green J. Stapleton M. R. Smith L. J. Artymiuk P. J. Kahramanoglou C. Hunt D. M. (2014). Cyclic-AMP and bacterial cyclic-AMP receptor proteins revisited: adaptation for different ecological niches. *Curr. Opin. Microbiol.* 18 1–7. 10.1016/j.mib.2014.01.003 24509484 PMC4005916

[B19] Groeneveld M. Weme R. G. Duurkens R. H. Slotboom D. J. (2010). Biochemical characterization of the C4-dicarboxylate transporter DctA from *Bacillus subtilis*. *J. Bacteriol.* 192 2900–2907. 10.1128/jb.00136-110 20363944 PMC2876488

[B20] Guo F. B. Xiong L. Teng J. L. Yuen K. Y. Lau S. K. Woo P. C. (2013). Re-annotation of protein-coding genes in 10 complete genomes of neisseriaceae family by combining similarity-based and composition-based methods. *DNA Res.* 20 273–286. 10.1093/dnares/dst009 23571676 PMC3686433

[B21] Han S. O. Inui M. Yukawa H. (2008). Effect of carbon source availability and growth phase on expression of *Corynebacterium glutamicum* genes involved in the tricarboxylic acid cycle and glyoxylate bypass. *Microbiology* 154 3073–3083. 10.1099/mic.0.2008/019828-19820 18832313

[B22] Heroven A. K. Dersch P. (2014). Coregulation of host-adapted metabolism and virulence by pathogenic yersiniae. *Front. Cell Infect. Microbiol.* 4:146. 10.3389/fcimb.2014.00146 25368845 PMC4202721

[B23] Howe E. A. Sinha R. Schlauch D. Quackenbush J. (2011). RNA-Seq analysis in MeV. *Bioinformatics* 27 3209–3210. 10.1093/bioinformatics/btr490 21976420 PMC3208390

[B24] Kanehisa M. Araki M. Goto S. Hattori M. Hirakawa M. Itoh M. (2008). KEGG for linking genomes to life and the environment. *Nucleic Acids Res.* 36 D480–D484. 10.1093/nar/gkm882 18077471 PMC2238879

[B25] Kawai S. Suzuki H. Yamamoto K. Inui M. Yukawa H. Kumagai H. (1996). Purification and characterization of a malic enzyme from the ruminal bacterium *Streptococcus bovis* ATCC 15352 and cloning and sequencing of its gene. *Appl. Environ. Microbiol.* 62 2692–2700. 8702261 10.1128/aem.62.8.2692-2700.1996 PMC168054

[B26] Kim D. S. Wi Y. M. Choi J. Y. Peck K. R. Song J. H. Ko K. S. (2011). Bacteremia caused by *Laribacter hongkongensis* misidentified as *Acinetobacter lwoffii*: report of the first case in Korea. *J. Korean Med. Sci.* 26 679–681. 10.3346/jkms.2011.26.5.679 21532861 PMC3082122

[B27] Kleijn R. J. Buescher J. M. Le Chat L. Jules M. Aymerich S. Sauer U. (2010). Metabolic fluxes during strong carbon catabolite repression by malate in *Bacillus subtilis*. *J. Biol. Chem.* 285 1587–1596. 10.1074/jbc.M109.061747 19917605 PMC2804316

[B28] Kong H. K. Law H. W. Liu X. Law C. O. Pan Q. Gao L. (2017). Transcriptomic analysis of laribacter hongkongensis reveals adaptive response coupled with temperature. *PLoS One* 12:e0169998. 10.1371/journal.pone.0169998 28085929 PMC5234827

[B29] Landete J. M. Garcia-Haro L. Blasco A. Manzanares P. Berbegal C. Monedero V. (2010). Requirement of the *Lactobacillus casei* MaeKR two-component system for L-malic acid utilization via a malic enzyme pathway. *Appl. Environ. Microbiol.* 76 84–95. 10.1128/aem.02145-2149 19897756 PMC2798650

[B30] Langmead B. Salzberg S. L. (2012). Fast gapped-read alignment with Bowtie 2. *Nat. Methods* 9 357–359. 10.1038/nmeth.1923 22388286 PMC3322381

[B31] Lau S. K. Lee L. C. Fan R. Y. Teng J. L. Tse C. W. Woo P. C. (2009). Isolation of *Laribacter hongkongensis*, a novel bacterium associated with gastroenteritis, from Chinese tiger frog. *Int. J. Food Microbiol.* 129 78–82. 10.1016/j.ijfoodmicro.2008.10.021 19033083

[B32] Lau S. K. Woo P. C. Fan R. Y. Lee R. C. Teng J. L. Yuen K. Y. (2007a). Seasonal and tissue distribution of *Laribacter hongkongensis*, a novel bacterium associated with gastroenteritis, in retail freshwater fish in Hong Kong. *Int. J. Food Microbiol.* 113 62–66. 10.1016/j.ijfoodmicro.2006.07.017 16996630

[B33] Lau S. K. Woo P. C. Fan R. Y. Ma S. S. Hui W. T. Au S. Y. (2007b). Isolation of *Laribacter hongkongensis*, a novel bacterium associated with gastroenteritis, from drinking water reservoirs in Hong Kong. *J. Appl. Microbiol.* 103 507–515. 10.1111/j.1365-2672.2006.03263.x 17714383

[B34] Leng N. Dawson J. A. Thomson V. Ruotti V. Rissman A. I. Smits B. M. G. (2013). EBSeq: an empirical bayes hierarchical model for inference in RNA-seq experiments. *Bioinformatics* 29 1035–1043. 10.1093/bioinformatics/btt087 23428641 PMC3624807

[B35] Li B. Dewey C. N. (2011). RSEM: accurate transcript quantification from RNA-Seq data with or without a reference genome. *BMC Bioinformatics* 12:323. 10.1186/1471-2105-12-323 21816040 PMC3163565

[B36] Liang W. Sultan S. Z. Silva A. J. Benitez J. A. (2008). Cyclic AMP post-transcriptionally regulates the biosynthesis of a major bacterial autoinducer to modulate the cell density required to activate quorum sensing. *FEBS Lett.* 582 3744–3750. 10.1016/j.febslet.2008.10.008 18930049 PMC2586060

[B37] Lorenz M. C. Fink G. R. (2001). The glyoxylate cycle is required for fungal virulence. *Nature* 412 83–86. 10.1038/35083594 11452311

[B38] McKinney J. D. Honer zu Bentrup K. Munoz-Elias E. J. Miczak A. Chen B. Chan W. T. (2000). Persistence of *Mycobacterium tuberculosis* in macrophages and mice requires the glyoxylate shunt enzyme isocitrate lyase. *Nature* 406 735–738. 10.1038/35021074 10963599

[B39] Meyer F. M. Jules M. Mehne F. M. Le Coq D. Landmann J. J. Gorke B. (2011). Malate-mediated carbon catabolite repression in *Bacillus subtilis* involves the HPrK/CcpA pathway. *J. Bacteriol.* 193 6939–6949. 10.1128/jb.06197-6111 22001508 PMC3232832

[B40] Mortazavi A. Williams B. A. McCue K. Schaeffer L. Wold B. (2008). Mapping and quantifying mammalian transcriptomes by RNA-Seq. *Nat. Methods* 5 621–628. 10.1038/nmeth.1226 18516045 PMC13303166

[B41] Mortera P. Espariz M. Suarez C. Repizo G. Deutscher J. Alarcon S. (2012). Fine-tuned transcriptional regulation of malate operons in *Enterococcus faecalis*. *Appl. Environ. Microbiol.* 78 1936–1945. 10.1128/aem.07280-7211 22247139 PMC3298141

[B42] Munoz-Elias E. J. McKinney J. D. (2005). Mycobacterium tuberculosis isocitrate lyases 1 and 2 are jointly required for in vivo growth and virulence. *Nat. Med.* 11 638–644. 10.1038/nm1252 15895072 PMC1464426

[B43] Myllykallio H. Liebl U. (2000). Dual role for cytochrome cbb3 oxidase in clinically relevant *proteobacteria*? *Trends Microbiol.* 8 542–543. 10.1016/s0966-842x(00)91831-6 11201260

[B44] Ni X. Sun J. Kong Q. Kong F. Brown M. Shen L. (2011). Isolation of *Laribacter hongkongensis* from little egrets (*Egretta garzetta*) in hangzhou. China. *Lett. Appl. Microbiol.* 52 465–467. 10.1111/j.1472-765X.2011.03024.x 21299577

[B45] Paluscio E. Caparon M. G. (2015). Streptococcus pyogenes malate degradation pathway links pH regulation and virulence. *Infect. Immun.* 83 1162–1171. 10.1128/iai.02814-2814 25583521 PMC4333477

[B46] Raja M. K. Ghosh A. R. (2014). Laribacter hongkongensis: an emerging pathogen of infectious diarrhea. *Folia Microbiol.* 59 341–347. 10.1007/s12223-013-0299-296 24481985

[B47] Shimizu K. (2013). Regulation systems of bacteria such as *Escherichia coli* in response to nutrient limitation and environmental stresses. *Metabolites* 4 1–35. 10.3390/metabo4010001 24958385 PMC4018673

[B48] Sigrist C. J. Cerutti L. de Castro E. Langendijk-Genevaux P. S. Bulliard V. Bairoch A. (2010). PROSITE, a protein domain database for functional characterization and annotation. *Nucleic Acids Res.* 38 D161–D166. 10.1093/nar/gkp885 19858104 PMC2808866

[B49] Soberon-Chavez G. Alcaraz L. D. Morales E. Ponce-Soto G. Y. Servin-Gonzalez L. (2017). The transcriptional regulators of the CRP family regulate different essential bacterial functions and can be inherited vertically and horizontally. *Front. Microbiol.* 8:959. 10.3389/fmicb.2017.00959 28620358 PMC5449483

[B50] Stulke J. Hillen W. (1999). Carbon catabolite repression in bacteria. *Curr. Opin. Microbiol.* 2 195–201. 10.1016/s1369-5274(99)80034-80034 10322165

[B51] Teng J. L. Woo P. C. Ma S. S. Sit T. H. Ng L. T. Hui W. T. (2005). Ecoepidemiology of *Laribacter hongkongensis*, a novel bacterium associated with gastroenteritis. *J. Clin. Microbiol.* 43 919–922. 10.1128/jcm.43.2.919-922.2005 15695706 PMC548085

[B52] Vereecke D. Cornelis K. Temmerman W. Jaziri M. Van Montagu M. Holsters M. (2002). Chromosomal locus that affects pathogenicity of *Rhodococcus fascians*. *J. Bacteriol.* 184 1112–1120. 10.1128/jb.184.4.1112-1120.2002 11807072 PMC134788

[B53] Wang Z. Y. Thornton C. R. Kershaw M. J. Debao L. Talbot N. J. (2003). The glyoxylate cycle is required for temporal regulation of virulence by the plant pathogenic fungus *Magnaporthe grisea*. *Mol. Microbiol.* 47 1601–1612. 10.1046/j.1365-2958.2003.03412.x 12622815

[B54] Winter S. E. Thiennimitr P. Winter M. G. Butler B. P. Huseby D. L. Crawford R. W. (2010). Gut inflammation provides a respiratory electron acceptor for *Salmonella*. *Nature* 467 426–429. 10.1038/nature09415 20864996 PMC2946174

[B55] Woo P. C. Lau S. K. Teng J. L. Que T. L. Yung R. W. Luk W. K. (2004). Association of Laribacter hongkongensis in community-acquired gastroenteritis with travel and eating fish: a multicentre case-control study. *Lancet* 363 1941–1947. 10.1016/s0140-6736(04)16407-16406 15194253

[B56] Woo P. C. Lau S. K. Teng J. L. Yuen K. Y. (2005). Current status and future directions for Laribacter hongkongensis, a novel bacterium associated with gastroenteritis and traveller’s diarrhoea. *Curr. Opin. Infect. Dis.* 18 413–419. 10.1097/01.qco.0000180162.76648.c9 16148528

[B57] Woo P. C. Lau S. K. Tse H. Teng J. L. Curreem S. O. Tsang A. K. (2009). The complete genome and proteome of *Laribacter hongkongensis* reveal potential mechanisms for adaptations to different temperatures and habitats. *PLoS Genet.* 5:e1000416. 10.1371/journal.pgen.1000416 19283063 PMC2652115

[B58] Xiong L. Teng J. L. Botelho M. G. Lo R. C. Lau S. K. Woo P. C. (2016). Arginine metabolism in bacterial pathogenesis and cancer therapy. *Int. J. Mol. Sci.* 17:363. 10.3390/ijms17030363 26978353 PMC4813224

[B59] Xiong L. Teng J. L. Watt R. M. Kan B. Lau S. K. Woo P. C. (2014). Arginine deiminase pathway is far more important than urease for acid resistance and intracellular survival in *Laribacter hongkongensis*: a possible result of arc gene cassette duplication. *BMC Microbiol.* 14:42. 10.1186/1471-2180-14-42 24533585 PMC3936950

[B60] Xiong L. Teng J. L. Watt R. M. Liu C. Lau S. K. Woo P. C. (2015). Molecular characterization of arginine deiminase pathway in *Laribacter hongkongensis* and unique regulation of arginine catabolism and anabolism by multiple environmental stresses. *Environ. Microbiol.* 17 4469–4483. 10.1111/1462-2920.12897 25950829

[B61] Xiong L. Yang Y. Ye Y. N. Teng J. L. Chan E. Watt R. M. (2017). *Laribacter hongkongensis* anaerobic adaptation mediated by arginine metabolism is controlled by the cooperation of FNR and ArgR. *Environ. Microbiol.* 19 1266–1280. 10.1111/1462-2920.13657 28028888

[B62] Yang S. Xu H. Wang J. Liu C. Lu H. Liu M. (2016). Cyclic AMP receptor protein acts as a transcription regulator in response to stresses in deinococcus radiodurans. *PLoS One* 11:e0155010. 10.1371/journal.pone.0155010 27182600 PMC4868304

[B63] Yuen K. Y. Woo P. C. Teng J. L. Leung K. W. Wong M. K. Lau S. K. (2001). *Laribacter hongkongensis* gen. nov., sp. nov., a novel gram-negative bacterium isolated from a cirrhotic patient with bacteremia and empyema. *J. Clin. Microbiol.* 39 4227–4232. 10.1128/jcm.39.12.4227-4232.2001 11724825 PMC88529

[B64] Zhang X. P. Ebright R. H. (1990). Identification of a contact between arginine-180 of the catabolite gene activator protein (CAP) and base pair 5 of the DNA site in the CAP-DNA complex. *Proc. Natl. Acad. Sci. U.S.A.* 87 4717–4721. 10.1073/pnas.87.12.4717 2162054 PMC54188

