# Supplementary material for: Malate-Dependent Carbon Utilization Enhances Central Metabolism and Contributes to Biological Fitness of Laribacter hongkongensis via CRP Regulation
Source: Front Microbiol. 2019 Aug 28;10:1991. doi: 10.3389/fmicb.2019.01991 (PMC6722228; doi:10.3389/fmicb.2019.01991)
Supplement: Supplementary file 1 [file Data_Sheet_1.docx]

**SUPPLEMENTARY MATERIAL**

**TABLE S1** Classiﬁcation of malate- and CRP- regulated genes according to KEGG pathway.

| Functional gene group (KEGG class) | | No. of genes | |  | No. of genes | |
| --- | --- | --- | --- | --- | --- | --- |
|  |  | Malate activated | Malate repressed |  | CRP activated | CRP repressed |
| Cellular Processes | | 4 | 5 |  | 0 | 0 |
|  | Cell growth and death | 0 | 0 |  | 0 | 0 |
|  | Transport and catabolism | 4 | 5 |  | 0 | 0 |
|  | Cell motility | 0 | 0 |  | 0 | 0 |
|  |  |  |  |  |  |  |
| Environmental Information Processing | | 2 | 5 |  | 2 | 1 |
|  | Membrane transport | 1 | 3 |  | 1 | 1 |
|  | Signal transduction | 1 | 2 |  | 1 | 0 |
|  |  |  |  |  |  |  |
| Genetic Information Processing | | 14 | 10 |  | 2 | 1 |
|  | Folding, sorting and degradation | 4 | 3 |  | 0 | 0 |
|  | Replication and repair | 3 | 3 |  | 1 | 0 |
|  | Transcription | 2 | 1 |  | 1 | 1 |
|  | Translation | 5 | 3 |  | 0 | 0 |
|  |  |  |  |  |  |  |
| Metabolism | | 305 | 203 |  | 18 | 8 |
|  | Global and overview maps | 185 | 40 |  | 6 | 3 |
|  | Amino acid metabolism | 21 | 69 |  | 4 | 2 |
|  | Nucleotide metabolism | 9 | 14 |  | 0 | 0 |
|  | Carbohydrate metabolism | 62 | 20 |  | 4 | 2 |
|  | Energy metabolism | 8 | 24 |  | 1 | 1 |
|  | Glycan biosynthesis and metabolism | 1 | 1 |  | 0 | 0 |
|  | Lipid metabolism | 5 | 14 |  | 0 | 0 |
|  | Metabolism of cofactors and vitamins | 8 | 13 |  | 1 | 0 |
|  | Metabolism of terpenoids and polyketides | 1 | 0 |  | 0 | 0 |
|  | Xenobiotics biodegradation and metabolism | 3 | 1 |  | 0 | 0 |
|  | Biosynthesis of secondary metabolites | 1 | 0 |  | 0 | 0 |
|  | Metabolism of other amino acids | 4 | 7 |  | 0 | 0 |
|  |  |  |  |  |  |  |
| Organismal Systems | | 2 | 1 |  | 0 | 0 |
|  | Environmental adaptation | 0 | 0 |  | 0 | 0 |
|  | Aging | 2 | 1 |  | 0 | 0 |
|  | Immune system | 0 | 0 |  | 0 | 0 |
|  |  |  |  |  |  |  |
| Unknown | | 161 | 134 |  | 3 | 5 |
|  | Function unknown | 141 | 125 |  | 3 | 5 |
|  | NA class | 20 | 9 |  | 0 | 0 |
|  |  |  |  |  |  |  |
| Total | | 491 | 358 |  | 25 | 15 |

**TABLE S2** Bacterial strains and plasmids used in this study.

| Strains or plasmids | Relative characteristics | Source or reference |
| --- | --- | --- |
| **Strains** |  |  |
| *E. coli* DH5α | Cloning host, *lacZ*^-^ | Invitrogen |
| *E. coli* S17-1(λ pir) | Donor strain for conjugation | Xiong et al., 2017 |
| *E. coli* SM10(λ pir) | Donor strain for conjugation | Xiong et al., 2015 |
| HLHK9 | Patient isolate, Cef^+^, Sm^+^ | Xiong et al., 2014 |
| HLHK9∆*crp* | HLHK9 derivative with *crp* deletion | This study |
| **plasmids** |  |  |
| PCRII-TOPO | Cloning vector; *ori lacZ* Km^+^ | Invitrogen |
| pCVD442 | Suicide plasmid; *R6K* *ori* *mob* *RP4* *bla* *sacB* | Xiong et al., 2014 |
| pVIK165 | Suicide plasmid containing a promoterless *gfp*, Km^+^ | BCCM-LMBP |
| pVIK165-P*crp* | pVIK165 fusion with the promoter region of *crp* gene | This study |

**TABLE S3** Primers used in this study.

| Primers | Sequence (5' to 3')^a^ |
| --- | --- |
| For mutagenesis of *crp* | |
| LPW30863 (*crp*-UF) | TC**GAGCTC**GACGAGTTCTTCGAACTC |
| LPW30864 (*crp*-UR) | ACCCGGATGCGGTTCTGGAACAGGGCAAA |
| LPW30865 (*crp*-DF) | TGTTCCAGAACCGCATCCGGGTGACCAAA |
| LPW30866 (*crp*-DR) | CTAG**TCTAGA**ACCGGGTGGCTGGACATG |
| LPW30867 (*crp*-INF) | CCAGGGAGAACTGGTTAC |
| LPW30868 (*crp*-INR) | CATGCGGTTGACCATTTC |
| For qRT-PCR | |
| LPW21629 (*arcA1*-F) | GTCACCCTCAATCCGATG |
| LPW21630 (*arcA1*-R) | CACCACACCTTCACCTTG |
| LPW21631 (*arcA2*-F) | CCGAAGTGGTGCGTGAATC |
| LPW21632 (*arcA2*-R) | TCTTGTTGGTGTAGGTGTTGC |
| LPW21633 (*argR*-F) | TCAGGCGTTATGGCTTCC |
| LPW21634 (*argR*-R) | GTGTCAGGGTGTCGTAGAG |
| LPW21635 (*rpoB*-F) | GTGCTGTTCGTCAATGAG |
| LPW21636 (*rpoB*-R) | TAGGTCGTAGGATTCTTCG |
| LPW22260 (*ureA*-F) | ATCTATTGCCTCGCCGAAGTG |
| LPW22261 (*ureA*-R) | AGTGCTGCCGCCGAAATC |
| LPW27093 (*fnr*-SYB-F) | TGAGCCGTGAAGAAATCG |
| LPW27094 (*fnr*-SYB-R) | GACCGTAATCCAGCCTTG |
| LPW32234 (*crp*-SYB-F) | ACCTGCTGGACTACATCG |
| LPW32235 (*crp*-SYB-R) | ATGCGGTTGACCATTTCG |
| LPW32236 (*maeB*-SYB-F) | GAACTGGTCAACAACGAGAAC |
| LPW32237 (*maeB*-SYB-R) | TCTTGCGGCTCACTTCAC |
| LPW32594 (*mdh*-SYB-F) | GCCTGTTGTTCCGCATTG |
| LPW32595 (*mdh*-SYB-R) | GTCTTCCAGTTCCATCATCAC |
| LPW32596 (*ppsA*_SYB-F) | CCGTCTGGGCAAGTATGTC |
| LPW32597 (*ppsA*_SYB-R) | AGCGAGAGGGTGTGGATG |
| LPW32598 (*capP*_SYB-F) | GCGAAGAAGAACCCTACC |
| LPW32599 (*capP*_SYB-R) | CGAATGGAACGGAATGTC |
| For constructing different promoter fragments | |
| LPW32629 (P*crp*-F) | C**GAGCTC**CGAGTTCTTCGAACTCTG |
| LPW32630 (P*crp*-R) | GC**TCTAGA**TCAAGCACAACGGTCAGG |
| LPW21098 (pVIK165-F2) | CCCTTAGAGCCTCTCAAAGC |
| LPW21026 (pVIK165-R2) | TGTTTCCTCCGAATTCGAGG |

^a^Restriction sites in the primer sequences appear in bold.

**TABLE S4** List of all differentially expressed genes (DEGS) with a FDR ≤ 0.01 and a │log_2_ ratio│ ≥ 1.0.
